# Impact of low-level laser therapy on orthodontic pain

**DOI:** 10.3389/fneur.2025.1666348

**Published:** 2025-11-11

**Authors:** Sylwia Jagła, Hanna Bielawska-Victorini, Krzysztof Woźniak

**Affiliations:** 1 Department of Maxillofacial Orthopaedics and Orthodontics, Collegium Medicum in Bydgoszcz, Nicolaus Copernicus University, Torun, Poland; 2 Department of Maxillofacial Orthopaedics and Orthodontics, Pomeranian Medical University, Szczecin, Poland

**Keywords:** pain, orthodontic appliances, low- level light therapy, low-level laser therapy, LLLT, pain management, pain measurement, fixed appliance

## Abstract

**Introduction:**

Pain is a primary deterrent to patient compliance in orthodontics. While pharmacological options exist, their systemic side effects warrant exploration of non-invasive alternatives. This study introduces Low-Level Laser Therapy (LLLT) as a localized, non-pharmacological intervention and evaluates its efficacy in mitigating pain during the critical initial phase of fixed appliance treatment.

**Methods:**

In this prospective controlled study, 60 patients (aged 14.6–37.1 years) were randomized into an LLLT group (*n* = 20) and a control group (*n* = 40). The LLLT group received daily photobiomodulation (670 nm, 4–6 J/cm^2^) for five consecutive days post-appliance placement. Pain was assessed daily using the Visual Analogue Scale (VAS), Laitinen Scale, and Verbal Rating Scale (VRS). Oral hygiene was evaluated using the Approximal Plaque Index (API).

**Results:**

LLLT significantly reduced pain perception. Pain peaked on day 2 for both groups, but the VAS score was lower in the LLLT group (4.35) than the control group (5.30). By day 4, the difference was highly significant (LLLT: 2.05 vs. Control:3.77; *p* < 0.0014). Furthermore, the LLLT group demonstrated significantly better oral hygiene, with a lower increase in API scores from T0 to T5 compared to the control group (*p* < 0.0003).

**Conclusion and application:**

LLLT is an effective and safe modality for reducing acute orthodontic pain. Its localized action, absence of systemic side effects, and positive influence on oral hygiene make it available clinical tool for improving patient comfort and compliance during orthodontic therapy. This study provides strong evidence supporting the integration of LLLT into routine orthodontic practice.

## 1 Introduction

Pain is one of the most frequently reported complaints among patients undergoing orthodontic treatment. The prevalence of pain during orthodontic therapy is estimated to range from 72 to 100% across a wide age spectrum (1, 2). Patient-reported discomfort, commonly referred to as “pain,” is frequently described in the literature using terms such as soreness, pressure, tension, strain, and hypersensitivity (2, 3). The mechanism of pain development during orthodontic treatment is strongly associated with the application of a specific force exerted on the tooth as a biological unit. The force applied to an orthodontic bracket is transmitted to the periodontal apparatus and alveolar bone, triggering a cascade of cellular, vascular, and immune responses. Local inflammatory mediators, including bradykinin and prostaglandins, play a role in stimulating pain receptors (2). Continuous pressure leads to the formation of ischemic areas, which in turn induce an inflammatory response and swelling of the periodontal tissues.

Importantly, this nociceptive input is integrated within the central nervous system, particularly via the trigeminal nerve pathways, which serve as the primary sensory conduit for the orofacial region. The trigeminal nerve (cranial nerve V) transmits pain signals from the periodontal ligament and surrounding tissues to the trigeminal ganglion and subsequently to higher-order brain centers, including the thalamus and somatosensory cortex, where pain is consciously perceived (4, 5). Additionally, the involvement of brainstem structures such as the trigeminal spinal tract nucleus plays a crucial role in modulating pain intensity and duration (6). This intricate neural processing underscores the functional connectivity between the stomatognathic system and the central nervous system, emphasizing that orthodontic pain is not merely a localized peripheral phenomenon but rather a complex neurosensory experience (7). The bidirectional relationship between these systems also suggests that emotional, cognitive, and autonomic factors can influence the perception of pain, further illustrating the multidimensional nature of orthodontic discomfort (8, 9).

Previous studies have extensively explored the intensity and progression of orthodontic pain over time, particularly within the first 7 days following the placement of a fixed appliance. It has been established that pain intensity gradually increases from the fourth hour post-placement, peaks at 24 h, and subsequently declines to baseline levels within 7 days (10).

The evaluation of available pain prevention methods in orthodontics remains a subject of ongoing discussion in the literature (11–16). The studies conducted in 2015 focused on identifying strategies to mitigate pain in orthodontic patients during the initial phase of treatment (13). Among the proposed interventions are the administration of analgesics, low-level laser therapy (LLLT), vibration therapy, behavioral therapy, transcutaneous electrical nerve stimulation (TENS), as well as the use of bite wafers, chewing gum, anesthetic gels, and orthodontic wax.

The dynamic advancement of medicine has been particularly evident in the widespread adoption of laser therapy. Lasers are utilized in various fields of medicine, enabling innovative treatment approaches and effective disease management. The analgesic properties of laser therapy, along with its ability to accelerate healing processes, have contributed to its extensive application in dentistry, including disciplines such as surgery, periodontology, endodontics, and orthodontics.

Low-Level Laser Therapy (LLLT), also referred to as photobiomodulation, has emerged as a promising non-invasive modality for managing orthodontic pain through its multifaceted interaction with neural and cellular mechanisms. On a peripheral level, LLLT reduces the production of pro-inflammatory mediators such as prostaglandins, bradykinin, and substance P, thereby decreasing nociceptor sensitization within the periodontal ligament (17, 18). At the same time, laser therapy enhances mitochondrial activity and ATP production in local tissues, accelerating cellular repair and reducing ischemia-induced inflammatory responses (19).

From a neurophysiological standpoint, LLLT influences the conduction velocity of Aδ and C fibers, modulating the transmission of nociceptive signals to the central nervous system (20). Furthermore, evidence suggests that laser therapy can impact neuronal membrane polarization and synaptic transmission, potentially altering central pain processing and perception within the trigeminal pathways (21). These neuromodulatory effects underline the therapeutic relevance of LLLT in the context of the bidirectional relationship between the stomatognathic and nervous systems. By modulating both local tissue inflammation and central sensory pathways, LLLT not only provides analgesia but may also contribute to improved neuroplastic adaptation during orthodontic treatment (22).

It is crucial to contextualize LLLT within the broader spectrum of light-based medical therapies, which leverage different mechanisms of action based on laser parameters and the presence of exogenous agents. While LLLT, or photobiomodulation, operates at low power densities to stimulate cellular processes without thermal damage, higher-energy applications can induce controlled thermal effects. This principle is harnessed in photothermal therapy (PTT), where agents like gold nanorods are used to absorb light and generate localized heat for therapeutic purposes, such as in cancer treatment (23, 24).

Another advanced modality is photodynamic therapy (PDT), which involves a photo sensitizer that, upon light activation, produces reactive oxygen species to induce targeted cell death. The versatility of PDT has been demonstrated in oncology, using novel agents like photoactive folic acid nanocomposites for targeted therapy (25). The combined use of nanoparticles and phototherapy represents a significant breakthrough in targeted medical treatments (26). While the present study focuses on the non-thermal, biostimulatory effects of LLLT for analgesia, understanding these alternative mechanisms is vital. It suggests future possibilities where, for instance, a mild photothermal or photodynamic approach could potentially be explored for other applications in orthodontics, such as accelerated tooth movement or enhanced disinfection, distinguishing the current study’s focus on pure photobiomodulation for pain relief.

Findings from numerous studies have positively evaluated the efficacy of low-level laser therapy (LLLT) in alleviating discomfort associated with orthodontic treatment (16, 27, 28). The perception of pain following orthodontic force application remains a key factor influencing patient cooperation, satisfaction, and treatment outcomes.

Given the growing interest in non- pharmacological methods of pain management in orthodontics, evaluating the efficacy of LLLT in this context appears both clinically relevant and timely. Therefore, this study was designed to investigate the effects of LLLT on perceived pain intensity and its subsequent impact on oral hygiene practices during the initial phase of fixed appliance orthodontic treatment.

Although the analgesic effect of low-level laser therapy (LLLT) in orthodontics has been previously investigated, the present study offers a novel perspective by simultaneously assessing pain intensity and oral hygiene status during the initial phase of fixed appliance treatment. Unlike many earlier studies, which focused solely on subjective pain perception, this research explores how pain modulation via LLLT may influence patients’ hygiene behaviors, using the Approximal Plaque Index (API) as a measurable clinical parameter. The study was conducted under routine clinical conditions in a mixed-age population, enhancing the external validity of the findings. By comparing the LLLT group with a non-intervention control group, we provide new insights into the natural course of post-orthodontic pain and the potential benefits of LLLT as a non-pharmacological adjunct in everyday orthodontic care.

## 2 Methods

Ethical approval was obtained prior to study initiation (approval no. BN-001/45/07, issued by the Bioethics Committee of the Pomeranian Medical University in Szczecin). All participants (or their legal guardians) provided written informed consent before inclusion in the study.

### 2.1 Eligibility criteria

A study involved 94 patients starting orthodontic treatment. After applying strict inclusion and exclusion criteria, 60 patients aged 14 to 37 years were selected. The inclusion criteria were as follows: informed consent for treatment and participation in the study, malocclusion qualifying for treatment with fixed edgewise appliances, complete medical documentation, including additional diagnostic records such as study models and radiographic images: panoramic and lateral cephalometric X-rays.

Exclusion criteria for patient participation in the study were as follows: pregnancy, malignant neoplasms, epilepsy, use of immunosuppressive drugs, use of photosensitizing medications, intellectual disability, mental illnesses and depressive disorders, cardiovascular diseases, bleeding tendency, gingival and periodontal diseases, ulcers and other inflammatory lesions within the oral cavity, temporomandibular joint disorders, chronic pain conditions, congenital anomalies, e.g., facial cleft defects.

To ensure the anonymity of study participants, each individual was assigned a randomly selected identification code. The group of 60 patients qualified for the study was randomly divided into two groups:

- LLLT (Low Level Laser Therapy) Group - 20 patients undergoing orthodontic therapy with fixed appliances in the initial phase of orthodontic treatment. This group underwent daily low-level laser therapy (LLLT) for five consecutive days following the placement of the fixed appliance.
- Control Group - 40 patients undergoing orthodontic therapy with fixed appliances in the initial phase of orthodontic treatment. No pain-relieving agents or procedures were applied in this group.

This pilot study enrolled a convenience sample of patients undergoing fixed orthodontic treatment at a private clinic. Although formal power analysis suggested a minimum of 64 participants for medium effect size detection, a smaller sample was used due to the exploratory nature of the trial and resource limitations. Patients were enrolled consecutively based on inclusion criteria until the predefined group sizes were reached.

This study is part of a larger research project evaluating non-pharmacological interventions for pain management during orthodontic treatment. Three groups were initially defined: LLLT (*n* = 20), vibration therapy (*n* = 20), and control (*n* = 40). The larger control group was intentionally designed to serve as a common reference for both experimental arms, increasing statistical power for baseline comparisons without intervention. In this article, only the LLLT and control groups are analyzed. The current sample size reflects the pilot nature of this study phase.

This study design prioritized the comparison of active Low-Level Laser Therapy (LLLT) with the natural course of pain resolution, as experienced by patients receiving standard care without analgesic intervention.

### 2.2 Low level laser therapy (LLLT)

In the LLLT group, biostimulatory laser therapy was performed using the MED-701 laser (Lasotronic, Switzerland). This device is a diode laser (InGaAlP) emitting a visible red light beam with a wavelength of 670 nm and a power output of < 350 mW. The device allows for adjustment of intensity levels, enabling precise energy dose selection depending on clinical needs. For this study, intensity level 3 was used, corresponding to 60% of the maximum power (approximately 180 mW). Laser therapy was applied using a contact technique, meaning the fiber-optic tip with a circular beam diameter of 7 mm remained in direct contact with the patient’s oral mucosa to minimize reflection and maximize energy transmission to underlying tissues. The therapy was conducted point-by-point over the mucosa in projection of the root apices of teeth included in the archwire system. The energy dose was determined based on the size and depth of the target tissue area, aiming for sufficient tissue penetration. A dose range of 4–6 J/cm^2^ was selected in line with current recommendations for biostimulatory purposes. The energy dose was calculated using the formula: E = P × t, where: E is energy in joules (J), P is power in watts (W), t is time in seconds (s). For this protocol: Power output (P): 180 mW (0.18 W), Time of application per point (t): 30 s, Calculated energy per point: 0.18 W × 30 s = 5.4 J, approximately 6 J/cm^2^. Laser application was performed once daily for five consecutive days, beginning immediately after bonding the fixed appliance. In total, five treatment sessions were conducted per patient. Importantly, both the operator and the patient wore protective safety goggles during each session to prevent accidental exposure to direct or reflected laser light. All procedures were performed by a licensed orthodontic specialist (DDS).

### 2.3 Algometric analysis

Each patient underwent an assessment of pain threshold in the tissues using a digital force algometer (DFA). The test was performed extraorally, at the projection of the masseter muscles near the mandibular angle, before appliance placement (T0). The pressure pain threshold was measured separately for the right and left sides. Additionally, an auxiliary measurement was conducted on the wrist of each participant.

During the examination, a constant pressure increment of 1.8 atm (1800 g) over 2 s was applied to the skin surface. Patients independently rated their perceived discomfort using a 100-mm Visual Analogue Scale (VAS). The measurement results were recorded in the study form.

### 2.4 Oral hygiene assessment

The oral hygiene level of the patients was assessed using the Approximal Plaque Index (API). A basic dental diagnostic set (mirror and probe) was used for the examination, supplemented with disclosing tablets or a plaque-disclosing solution. The presence (+) or absence (−) of plaque was recorded.

Measurements were taken on the day of fixed light-wire appliance placement (T0) and again on the fifth day after placement (T5) for each participant.

### 2.5 Survey assessment of pain and hygiene habits

All participants completed an anonymous questionnaire on the day of appliance placement before (T0) and after (T1) placement, as well as on four subsequent days (T2, T3, T4, T5). The survey consisted of two parts:

1. Assessment of Perceived Pain

- Visual Analogue Scale (VAS)
  The scale’s starting point was described as “no pain,” while the endpoint represented “the worst pain imaginable” (29).
- Laitinen Questionnaire
  The Laitinen Scale is a subjective pain intensity measurement ranging from 0 to 4, where 0 indicates no pain and 4 represents maximum pain intensity. The questionnaire evaluated the severity of the pain experienced (30).
- Verbal Rating Scale (VRS)
  The Verbal Rating Scale (VRS) was used to assess pain intensity using descriptive terms. A six-level verbal scale from the Melzack questionnaire was applied (31).

2. Evaluation of Hygiene Habits During Orthodontic Treatment

The second part of the questionnaire examined changes in daily oral hygiene habits during fixed light-wire appliance therapy. It focused on the level of discomfort experienced during routine oral hygiene procedures.

### 2.6 Statistical analysis

The results obtained in the study were statistically analyzed. The Shapiro–Wilk test was used to verify the hypothesis of normality in the distribution of the variable.

To test the hypothesis regarding the presence or absence of differences between mean values for independent variables, the Kruskal- Wallis rank test, the median test, and the Mann–Whitney U test were applied. For dependent variables, the hypothesis of differences between mean values was tested using Friedman’s two-way analysis of variance (ANOVA) and Wilcoxon’s signed-rank test.

The Pearson product–moment correlation coefficient (r) and Spearman’s rank correlation coefficient (r) were used to assess the correlation between variables. To evaluate associations between categorical or qualitative variables, the chi-square (*χ*^2^) independence test, the Yates-corrected chi-square test, or Fisher’s exact test were employed.

Advanced multivariate methods were applied to assess the probability of variable differentiation based on specific qualitative predictors and their discriminative functions. The differentiation of multiple variables within groups defined by qualitative factors was analyzed using one-way analysis of variance (ANOVA/ANCOVA) models. The differentiation of multiple variables within groups defined by qualitative factors or their interactions was examined using multivariate analysis of variance (MANOVA/MANCOVA) models.

For all hypothesis testing, a significance level of *p* = 0.05 was adopted. Statistical analyses were performed using Statistica (StatSoft) software.

## 3 Results

### 3.1 Age structure of the study participants

**Figure 1:**
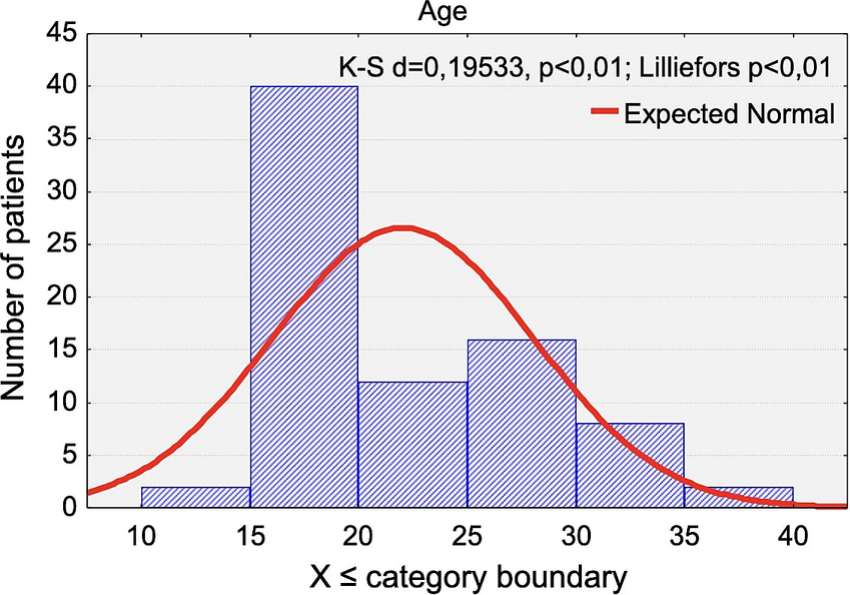
Distribution of the chronological age of the study participants.

The age distribution of the participants is presented in Figure 1. The mean age was 22.03 years (range: 14.6 to 37.1 years) and did not differ significantly between women (22.72 years) and men (20.77 years) (*p* < 0.4172).

The mean age within each group was as follows: control group: 21.22 years (women: 21.65, men:20.51) and LLLT group: 23.70 years (women: 25.46, men: 19.60). LLLT group: 23.70 years (women: 25.46, men: 19.60).

### 3.2 Pain threshold assessment using algometry

The results of algometric assessment before the placement of fixed appliances (T0), considering three measurement points (right mandibular angle, left mandibular angle, and wrist), are presented in Tables 1, 2.

**Table 1:** Distribution of algometric measurements in the control group before appliance placement (T0).

Location	*n*	*x̅*	−95.00% CI	+95.00% CI	Me	Min.	Maks.	SD	SEM
Mandibular Angle R	40	3.90	3.39	4.41	4.00	1.00	8.00	1.58	0.37
Mandibular Angle L	40	4.08	3.57	4.58	4.00	1.00	8.00	1.59	0.37
Wrist	40	0.60	0.33	0.87	0.00	0.00	3.00	0.84	0.37

n, number of participants (sample size); x̄, arithmetic mean; –95.00% CI/+95.00% CI, lower and upper bounds of the 95% confidence interval; M, median; Min./Max., minimum/maximum value; SD, standard deviation; SEM, standard error of the mean.

**Table 2:** Distribution of algometric measurements in the LLLT group before appliance placement (T0).

Location	*n*	*x̅*	−95.00% CI	+95.00% CI	Me	Min.	Maks.	SD	SEM
Mandibular Angle R	20	4.50	3.68	5.32	4.50	2.00	8.00	1.76	0.51
Mandibular Angle L	20	4.30	3.47	5.13	4.00	2.00	8.00	1.78	0.51
Wrist	20	0.75	0.32	1.18	0.50	0.00	3.00	0.91	0.51

n, number of participants (sample size); x̄, arithmetic mean; –95.00% CI/+95.00% CI, lower and upper bounds of the 95% confidence interval; M, median; Min./Max., minimum/maximum value; SD, standard deviation; SEM, standard error of the mean.

Pain intensity values assessed using the Visual Analogue Scale (VAS) ranged from 0 to 8.

There was no statistically significant difference at the right mandibular angle for both groups (*p* < 0.2203). There was no statistically significant difference at the left mandibular angle for both groups (*p* < 0.5388). There was no statistically significant difference at the wrist for both groups (*p* < 0.3457).

### 3.3 Oral hygiene assessment

**Figure 2:**
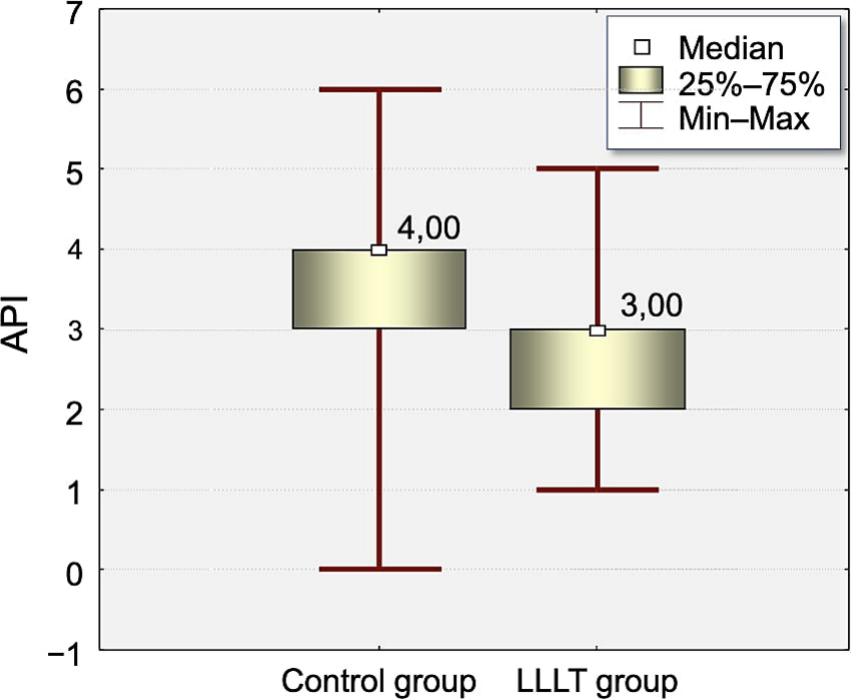
Distribution of Approximal Plaque Index (API) values measured on the day of appliance placement (T0) of the study in the Control and LLLT groups.

**Figure 3:**
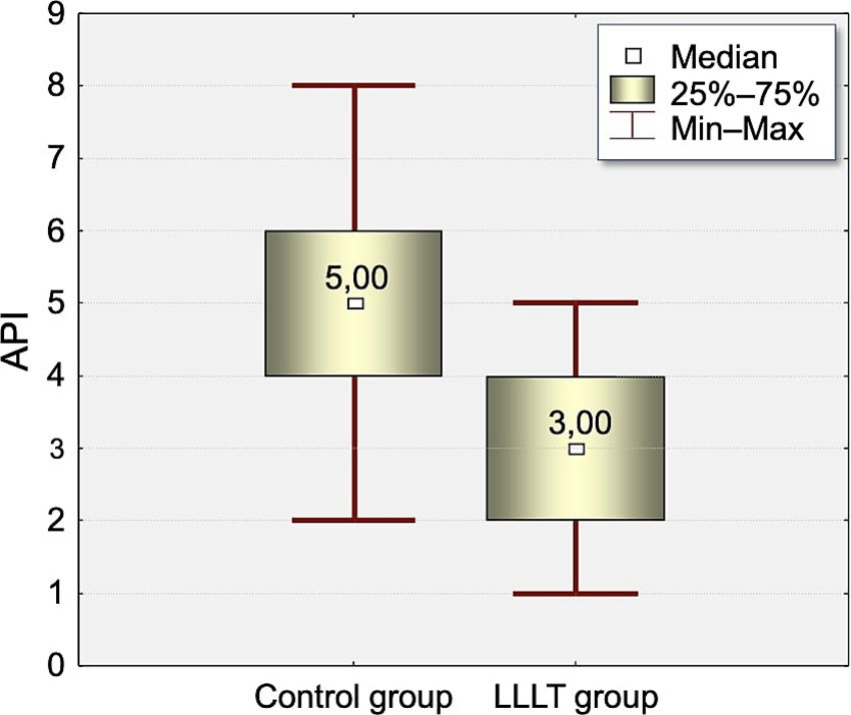
Distribution of Approximal Plaque Index (API) values measured on the fifth day (T5) of the study in the Control and LLLT groups.

The distribution of Approximal Plaque Index (API) values at the time of appliance placement (T0) and on the fifth day after appliance placement (T5) is presented in Tables 3, 4 and Figures 2, 3.

**Table 3:** Distribution of Approximal Plaque Index (API) values on the day of appliance placement (T0) and on the fifth day (T5) in the Control group.

Gender	*n*	*x̅*	−95.00% CI	+95.00% CI	M	Min.	Max.	SD	SEM
API /T0	40	3.75	3.35	4.14	4.00	0.00	6.00	1.23	0.19
API /T5	40	4.87	4.38	5.36	5.00	2.00	8.00	1.53	0.24

n, number of participants (sample size); x̄, arithmetic mean; –95.00% CI/+95.00% CI, lower and upper bounds of the 95% confidence interval; M, median; Min./Max., minimum/maximum value; SD, standard deviation; SEM, standard error of the mean.

**Table 4:** Distribution of Approximal Plaque Index (API) values on the day of appliance placement (T0) and on the fifth day (T5) in the LLLT group.

Gender	*n*	*x̅*	−95.00% CI	+95.00% CI	Me	Min.	Maks.	SD	SEM
API /T0	20	2.65	2.18	3.11	3.00	1.00	5.00	0.98	0.22
API /T5	20	3.20	2.56	3.83	3.00	1.00	5.00	1.36	0.30

n, number of participants (sample size); x̄, arithmetic mean; –95.00% CI/+95.00% CI, lower and upper bounds of the 95% confidence interval; M, median; Min./Max., minimum/maximum value; SD, standard deviation; SEM, standard error of the mean.

There was statistically significant difference between groups in API index on T0 and T5.

At T0, the API index was significantly higher in the control group (3.75) compared to the LLLT group (2.65, *p* < 0.0005). This difference increased on T5 (4.87 vs. 3.2, *p* < 0.0003).

### 3.4 Pain intensity (VAS)

Statistical analysis of VAS pain scores revealed slightly higher pain levels in men (3.84) compared to women (3.63, *p* < 0.5822).

A multivariate analysis of variance (MANOVA) confirmed significant differences (*p* < 0.0032) in mean pain intensity across groups.

Gender was not a determining factor in pain intensity across groups (*p* < 0.7785).

#### 3.4.1 Effect of archwire diameter on pain intensity

According to prior knowledge, the diameter of the initial archwire may significantly affect pain intensity (0.012-inch archwire: Mean pain intensity: 3.45; 0.014-inch archwire: Mean pain intensity: 3.98) (*p* < 0.3544).

**Figure 4:**
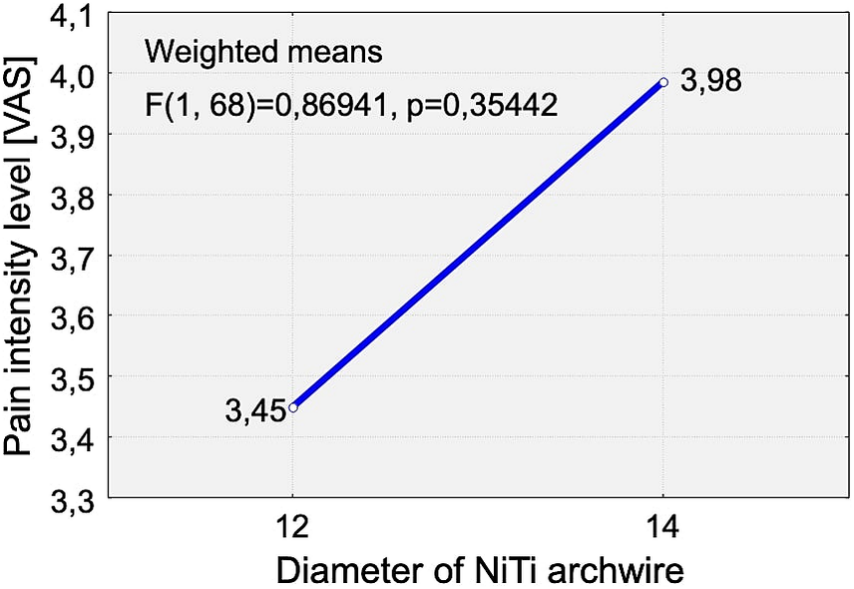
Distribution of pain intensity changes according to the Visual Analogue Scale [VAS scale] in relation to the diameter of the initial archwire used at appliance placement. Two wire sizes were analyzed: 0.012-inch NiTi and 0.014-inch NiTi. Each point represents the average pain intensity reported by participants for a given wire diameter.

The archwire diameter was not a determinant of pain intensity across groups (*p* < 0.8920, Figure 4).

#### 3.4.2 VAS pain intensity over time

**Figure 5:**
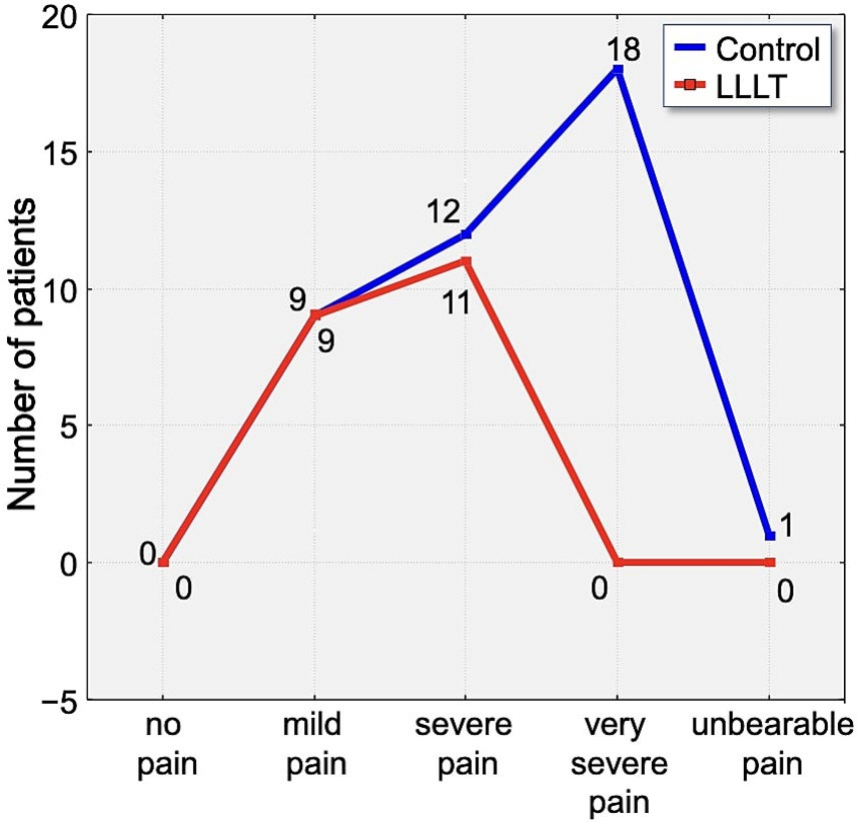
Distribution of pain intensity changes according to the Visual Analogue Scale [VAS scale] over time in the study groups. The graph presents the evolution of pain intensity in the Control (blue line) and LLLT (red line) groups over the 5-day observation period (T1–T5).

A detailed analysis of changes in VAS pain intensity revealed a consistent pattern across the observation period. On Day 1 (T1), pain intensity was significantly lower in the LLLT group (3.25) compared to the control group (4.53), with statistical significance (*p* < 0.0384). On the second day (T2), pain levels peaked in both groups. Although the difference did not reach statistical significance (*p* = 0.0542), the mean pain score remained notably lower in the LLLT group (4.35) compared to the control group (5.30). On Day 3 (T3), a significant reduction in pain was again observed in the LLLT group (3.40) compared to the control group (5.0), with *p* < 0.0082. This favorable trend continued on Day 4 (T4), with pain intensity significantly lower in the LLLT group (2.05) than in the control group (3.77), reaching strong statistical significance (*p* < 0.0014). Finally, on Day 5 (T5), the pattern persisted, with significantly reduced pain in the LLLT group (1.65) relative to the control group (2.65), again with *p* < 0.0014 (Figure 5).

### 3.5 Laitinen pain scale analysis

**Figure 6:**
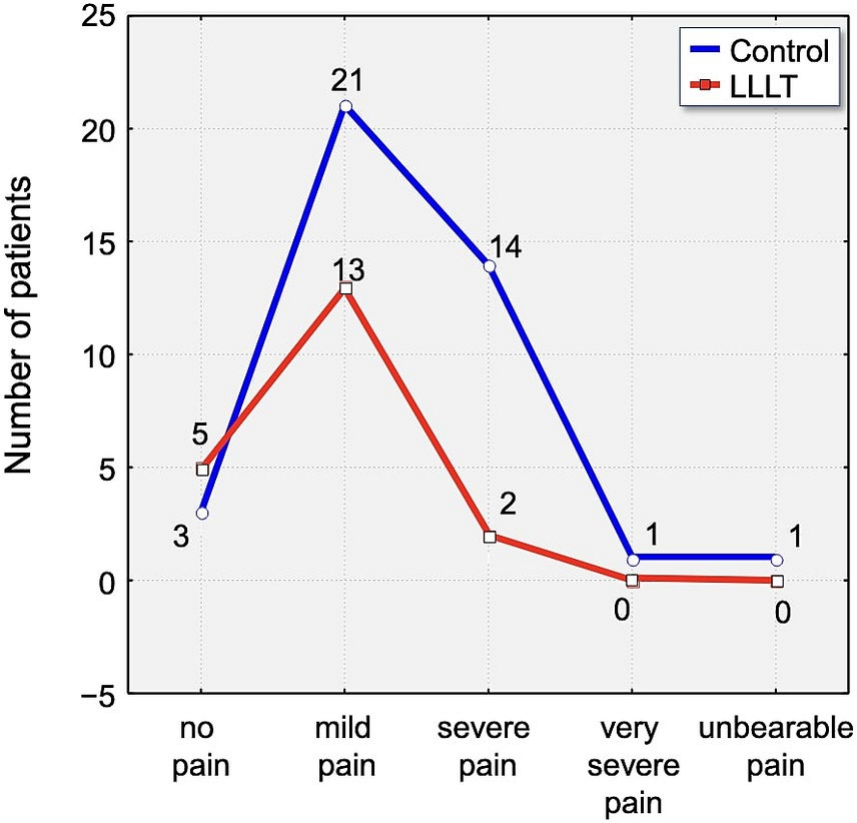
Distribution of pain intensity according to the Laitinen scale on Day 1 (T1). The graph shows the number of patients in the Control (blue line) and LLLT (red line) groups reporting different levels of pain, from “no pain” to “unbearable pain.” A clear shift towards lower pain intensities is visible in the LLLT group.

Assessment of pain using the Laitinen scale revealed notable differences in pain intensity between the study group over the observation period. On the first day following appliance placement, 70% of participants in the control group reported experiencing severe or very severe pain. In contrast, 65% of individuals in the LLLT group reported only mild pain, indicating a clear disparity in perceived discomfort (Figure 6).

**Figure 7:**
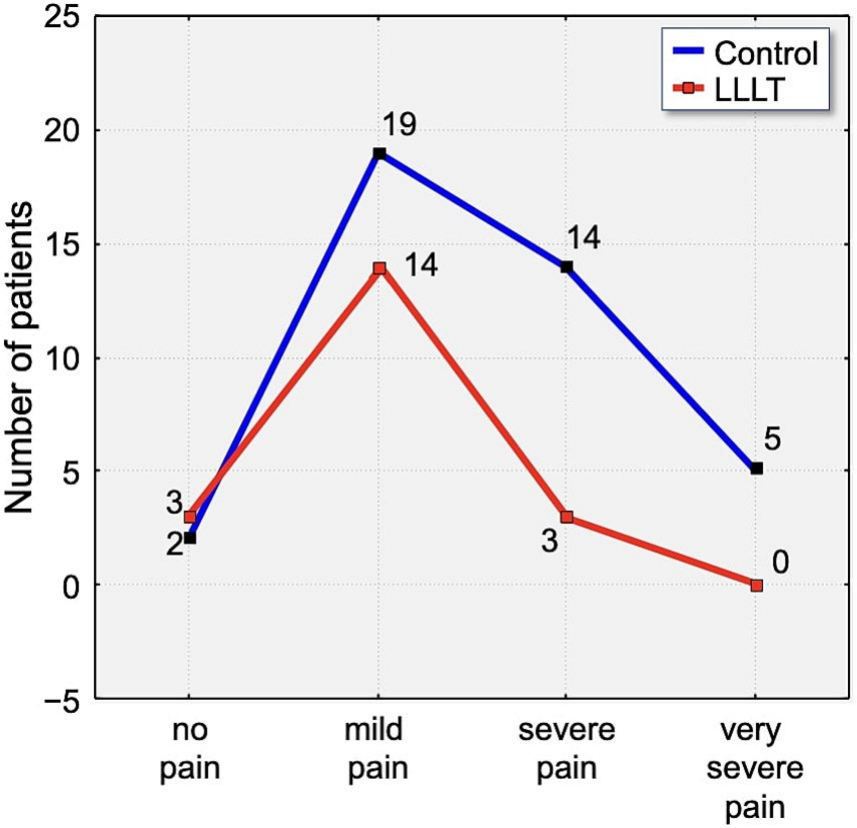
Distribution of pain intensity according to the Laitinen scale on Day 2 (T2). The graph shows the number of patients in the Control (blue line) and LLLT (red line) groups reporting different levels of pain, from “no pain” to “unbearable pain.” A clear shift towards lower pain intensities is visible in the LLLT group.

By the second day, 46.25% of all participants reported severe pain. Within this subset, 30% of control group participants experienced pain of severe intensity, compared to 55% in the LLLT group. Although a higher proportion of severe pain was reported in the LLLT group on this day, it is important to note that 2.5% of participants in the control group described their pain as unbearable, whereas no such reports were recorded in the LLLT group (Figure 7).

**Figure 8:**
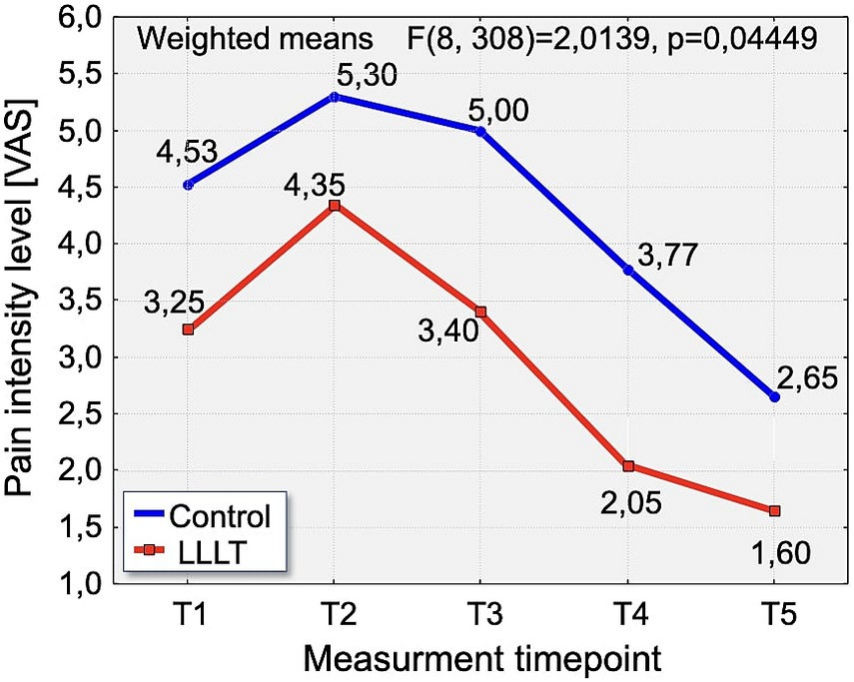
Distribution of pain intensity according to the Laitinen scale on Day 3 (T3). The graph shows the number of patients in the Control (blue line) and LLLT (red line) groups reporting different levels of pain, from “no pain” to “unbearable pain.” A clear shift towards lower pain intensities is visible in the LLLT group.

On the third day, a small percentage of participants (6.25%) reported complete absence of pain. Notably, none of the participants in the LLLT group experienced either very severe or unbearable pain. In contrast, 30% of control group members reported very severe pain, and 2.5% indicated their pain was unbearable (Figure 8).

**Figure 9:**
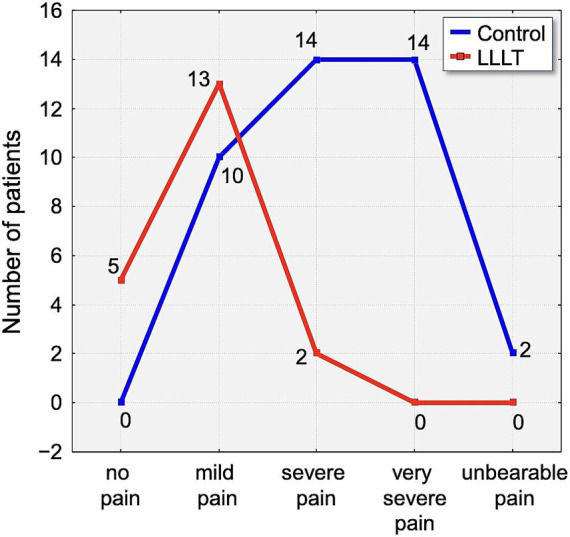
Distribution of pain intensity according to the Laitinen scale on Day 4 (T4). The graph shows the number of patients in the Control (blue line) and LLLT (red line) groups reporting different levels of pain, from “no pain” to “unbearable pain.” A clear shift towards lower pain intensities is visible in the LLLT group.

Statistically significant differences emerged again on the fourth day (T4). Complete absence of pain was reported by 5% of the control group and by 15% of the LLLT group. Mild pain was the most frequently reported intensity level on this day, accounting for 60% of responses across the study population (Figure 9).

**Figure 10:**
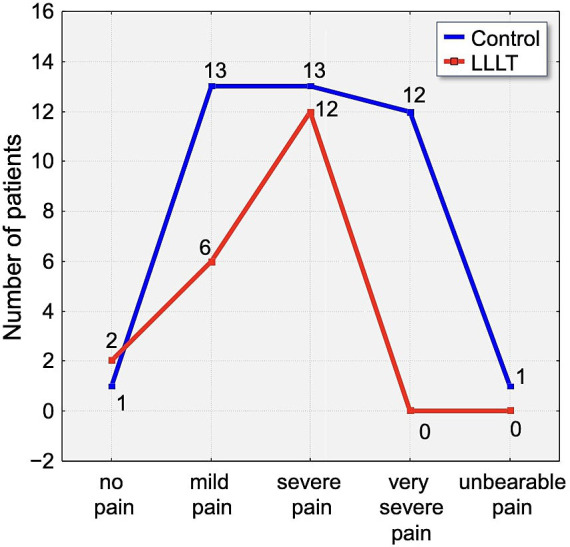
Distribution of pain intensity according to the Laitinen scale on Day 5 (T5). The graph shows the number of patients in the Control (blue line) and LLLT (red line) groups reporting different levels of pain, from “no pain” to “unbearable pain.” A clear shift towards lower pain intensities is visible in the LLLT group.

By the fifth day (T5), pain had further subsided. A total of 12.5% of participants reported no pain at all, with 7.5% from the control group and 25% from the LLLT group reporting complete relief. These findings support the consistent trend of reduced pain severity in patients treated with Low-Level Laser Therapy over the five-day observation period (Figure 10).

### 3.6 Verbal rating scale (VRS) pain intensity analysis

The history of pain intensity changes according to the Verbal Rating Scale (VRS). The analysis of pain intensity on the first day after fixed appliance placement (T1) in the study groups revealed statistically significant differences between the groups (*p* < 0.0061). Notably, complete absence of pain was reported by only one participant, who belonged to the control group. In contrast, 15% of individuals in the Low-Level Laser Therapy (LLLT) group experienced only mild pain. A considerable proportion of participants in the control group—47.5%—reported experiencing either severe or unbearable pain on Day 1, whereas the majority of individuals in the LLLT group - 80% - described their discomfort as mild to moderate.

On the second day, no participants in any group reported a complete absence of pain. Mild discomfort was noted in just one case within the control group. Reports of mild pain were observed in 5% of control group participants, 30% of those in the LLLT group, and in 10% of all study participants overall. Moderate pain was recorded in 20% of the control group and 40% of the LLLT group. Severe pain remained most prevalent in the control group, affecting 70% of its participants, compared to 30% in the LLLT group, a difference that was also statistically significant (*p* < 0.0018).

On the third day following appliance placement, the pattern of pain intensity continued to show marked differences between the study groups. Mild pain was reported by 15% of participants in the control group and 25% in the LLLT group. Severe pain persisted in 50% of the control group, while only 15% of participants in the LLLT group reported pain of this intensity, a statistically significant difference (*p* < 0.0211). Notably, while half of the control group continued to experience severe pain, half of the participants in the LLLT group described their discomfort as moderate.

By the fourth day, a general trend toward pain reduction was evident, although differences between the groups remained significant. Mild discomfort was noted by 2.5% of participants in the control group and by 15% in the LLLT group. Mild pain was more frequently reported in both groups, affecting 30% of the control group and 60% of the LLLT group. The most substantial divergence on Day 4 concerned moderate pain intensity, which was reported by 50% of the control group and only 20% of the LLLT group, a difference reaching statistical significance (*p* < 0.0067).

On the fifth day, complete absence of pain was reported by 5% of individuals in the control group and by 10% in the LLLT group. Mild pain was experienced by 37.5% of participants in the control group and by 50% of those in the LLLT group. As was the case on Day 4, the most prominent difference was observed in the prevalence of moderate pain. While 42.5% of the control group still reported moderate pain, only 10% of the LLLT group did so, a statistically significant disparity (*p* < 0.0039), further supporting the analgesic efficacy of low-level laser therapy during the initial phase of orthodontic treatment.

## 4 Discussion

Fear and anxiety are recognized modulators of both acute and chronic pain perception. As noted in previous research, individuals with high dental anxiety tend to overestimate and more intensely remember previous pain experiences, which in turn amplifies their future pain expectations (32).

Both in our own research and in reports from multiple other authors, pain symptoms following the initiation of orthodontic treatment with fixed appliances gradually increase within the first 24 h and subsequently decrease over the following 7 days (33–38).

In our study, no age restrictions were applied, and the sample consisted of 60 individuals aged 14–37 years. Both actively growing individuals and adults were included, allowing for a broader assessment of pain occurrence across different age groups.

Research by Krukemeyer et al. (39) on pain intensity during orthodontic treatment demonstrated that the vast majority of orthodontic patients experience pain, with 58.5% reporting the most intense discomfort within the first few days following an appointment. Pain assessment was conducted using a custom five-point scale designed to measure pain occurrence during treatment.

In our study, multiple pain scales were used, allowing not only for the determination of pain occurrence but also for the assessment of discomfort intensity. The inseparable association between fixed orthodontic treatment and pain formation has prompted investigations into various pain-relief methods and therapies to mitigate associated discomfort. According to numerous researchers, nonsteroidal anti-inflammatory drugs (NSAIDs) administered prior to orthodontic procedures have demonstrated significant analgesic effects during orthodontic treatment. Given that pain arises primarily from an inflammatory response, NSAID administration has been shown to inhibit the overproduction of prostaglandins in tissues, thereby reducing inflammation-related discomfort.

Bernhardt et al. confirmed the efficacy of administering 400 mg of ibuprofen 1 h before orthodontic separator placement, with statistically significant pain reduction observed from the second hour up to 24 h post-procedure, compared to a placebo group (10). Similarly, Patel et al. demonstrated the effectiveness of ibuprofen administration 1 h before orthodontic treatment and at three and 7 h post-procedure. However, naproxen sodium and acetaminophen did not provide satisfactory pain relief (40).

Studies by Polat and Karaman (41) also confirmed the effectiveness of NSAIDs in reducing orthodontic pain. Pain intensity across all parameters, except for biting, was lower in the NSAID group. However, the authors emphasized potential adverse effects of NSAIDs, including gastric and duodenal ulcers, bleeding disorders, kidney failure, asthma, allergies, hypertension, congestive heart failure, and atherosclerosis (41). These limitations should be considered when deciding on pharmacological analgesia.

NSAIDs exert a systemic analgesic effect, which limits their routine use. Moreover, literature reports suggest a controversial impact of pharmacological agents on orthodontic tooth movement (2). Nonetheless, NSAIDs remain the most commonly used and widely accepted pain management strategy. Given the adverse effects and potential risks associated with NSAIDs, our study focused on non-invasive, localized pain-relief methods that do not negatively affect the entire body. Our results demonstrated a significant reduction in pain levels in groups where low-level laser therapy (LLLT) was applied.

Pain intensity was assessed on the day of fixed appliance placement and for four consecutive days thereafter, following the methodologies of previous studies Ngan et al. (37), Scheurer et al. (38), Abdelrahman et al. (33), Asiry et al. (34), Erdinç and Dinçer (35). These studies have demonstrated that pain gradually increases within the first 24 h, peaks on the second day, and begins to decline from the third day onwards. This trend was also observed in our study, in both the control and experimental groups.

To ensure an accurate assessment of pain intensity, we separately evaluated the phototherapy group and the control group, which did not receive any pain-relief intervention. Participants were not asked to compare pain levels on different sides of the dental arch, as pain perception can broadly affect overall well-being, potentially leading to unreliable results.

Tortamano et al. (42) investigated the effectiveness of phototherapy in reducing pain after initial orthodontic archwire placement. The study utilized a Ga-Al-As diode laser (wavelength: 830 nm) and included 60 participants, divided into an experimental, placebo, and control group.

Pain levels were monitored for seven consecutive days, and results demonstrated that patients who underwent laser biostimulation experienced lower pain intensity and shorter pain duration than those in the placebo or control groups. However, LLLT did not affect the time of pain onset or the peak discomfort period (24–48 h post-procedure) (42).

Our findings align with those of Tortamano et al. (42) and other researcher (37), confirming similar patterns in pain perception following orthodontic appliance placement. Pain onset was observed on the first day in both groups; however, it was significantly lower in the group treated with Low-Level Laser Therapy (LLLT) compared to the control group. The highest intensity of pain occurred on the second day in both groups, yet the pain levels remained consistently lower in the LLLT group. From the third day onward, a gradual decrease in pain was noted in both cohorts, with the LLLT group continuing to report significantly reduced pain levels throughout the observation periodThese results support the use of continued LLLT applications beyond a single session for effective pain management in orthodontic patients. In our study, an InGaAlP diode laser (gallium-indium-aluminum-phosphide) with a wavelength of 670 nm was used. The emitted red light falls within the visible spectrum, similar to devices used by other researchers. According to Li et al. (43), who conducted a systematic review on LLLT applications, LLLT has not yet been established as a standard treatment for orthodontic pain relief, due to variability in laser system specifications and application protocols.

Numerous researchers have emphasized that unpleasant pain sensations during orthodontic treatment can negatively influence patients’ oral hygiene behaviors and may even lead to unfavorable dietary modifications. Pain-induced discomfort often results in reduced motivation to perform routine oral hygiene procedures, such as thorough brushing and interdental cleaning. A particularly noteworthy finding of this study is the significant improvement in oral hygiene, as measured by the API, in the LLLT group compared to the control group. This provides quantitative evidence for a clinically observed phenomenon: pain is a direct barrier to effective mechanical plaque removal. The discomfort associated with brushing and flossing around newly placed orthodontic appliances can lead to avoidance behavior, resulting in plaque accumulation and gingival inflammation. By effectively mitigating pain through photobiomodulation, LLLT appears to break this cycle. Patients experiencing less discomfort are more likely to maintain their oral hygiene routines, as reflected by the lower API scores. This indirect benefit of LLLT is of profound clinical importance, as poor hygiene can lead to complications such as gingivitis, decalcification, and caries, potentially compromising the overall success of orthodontic treatment. Therefore, LLLT may not only improve patient comfort but also contribute to better periodontal health and treatment outcomes by facilitating patient compliance with hygiene protocols (44, 45).

LLLT is a non-invasive, localized therapy that provides targeted pain relief at the site of application. Unlike pharmacological agents traditionally used for pain management, LLLT offers several notable advantages. It does not produce systemic side effects, does not interfere with orthodontic tooth movement, and is considered safe for long-term use. Moreover, it can be applied repeatedly without posing cumulative risks to the patient. Thus, LLLT presents a promising alternative to conventional pharmacological pain management strategies in orthodontic treatment.

Pain is an inherently subjective experience. Consequently, any attempt to objectively quantify it is fraught with challenges. The significant margin of error in pain research stems not primarily from methodological limitations, but from the individual and perceptual nature of pain itself.

Orthodontic treatment may influence both the pressure pain thresholds measured using algometry and patients’ attitudes toward treatment. This is particularly important given that the success of orthodontic therapy depends heavily on patient cooperation and motivation, which are closely linked to pain perception and psychological readiness (46). Psychological characteristics of patients may significantly affect not only their perception of pain but also their overall response to orthodontic treatment (47). Given the complex nature and widespread occurrence of pain associated with orthodontic treatment, it appears essential to conduct studies that allow for a more in-depth understanding of this issue.

In the present study, several pain assessment scales were utilized to evaluate the intensity of pain symptoms. To comprehensively capture the range and severity of patients’ pain experiences, the Visual Analogue Scale (VAS), Laitinen Questionnaire, and Verbal Rating Scale (VRS) were employed. Furthermore, the study incorporated an objective analysis using algometric examination.

In order to avoid the use of analgesics-despite their well-documented efficacy in reducing orthodontic pain, but also due to their broad spectrum of adverse effects and potential side effects—this study focused on non-invasive methods that act locally and do not negatively impact the entire body.

The biostimulatory laser is a specialized medical device intended for use exclusively by trained healthcare professionals under appropriately controlled conditions. Strict safety protocols must be followed to protect both the operator and the patient undergoing therapy. The effects of laser treatment are multifaceted, primarily leading to a reduction of inflammation in irradiated tissues. It also exerts a wide range of therapeutic effects—not only alleviating pain but also promoting healing processes. The laser has been shown to reduce the production of inflammatory mediators. Therefore, its analgesic effect should be considered a secondary benefit of laser biostimulation, rather than its primary therapeutic goal (*primus omnium*). This gives it a distinct advantage over pharmacological agents commonly used to manage pain symptoms.

Despite the promising results, this study has certain limitations that should be acknowledged. The relatively small sample size may limit the generalizability of the findings and reduce the statistical power to detect more subtle effects of LLLT. Additionally, the short duration of follow-up focused solely on the acute pain response may not fully capture the long-term impact of LLLT on pain modulation or oral hygiene behaviors during the entire course of orthodontic treatment. Future research should involve larger and more diverse patient populations, as well as extended observation periods, to validate these results and further explore the clinical benefits and optimal parameters of LLLT in orthodontics.

## 5 Conclusion

This study provides robust evidence that daily application of LLLT (670 nm) during the first 5 days of orthodontic treatment is a highly effective, non-invasive strategy for mitigating pain. The therapy not only significantly reduced perceived pain intensity, with peak pain levels remaining lower and resolving faster than in the control group, but also led to a measurable improvement in oral hygiene. This dual benefit underscores the clinical utility of LLLT in enhancing both patient comfort and compliance, critical factors for successful orthodontic outcomes. Future Perspectives: While this study confirms the analgesic efficacy of photobiomodulation, the broader field of phototherapy offers exciting future avenues. For severe or persistent orthodontic pain, a more potent intervention might be required. Future research could explore a mild photothermal approach, potentially using biocompatible nanoparticles to enhance light absorption and generate a controlled, localized temperature increase that could more effectively block nociceptive pathways. Furthermore, the use of a photosensitizer in a photodynamic therapy (PDT) protocol could be investigated, not for pain, but for its antimicrobial properties to manage plaque and gingivitis around orthodontic brackets. Investigating these advanced modalities, alongside optimizing LLLT protocols (e.g., determining the minimum effective number of sessions), will be crucial steps in fully harnessing the power of light-based therapies in orthodontics.

## Data Availability

The original contributions presented in the study are included in the article/supplementary material, further inquiries can be directed to the corresponding author.
